# Stem cells and cancer immunotherapy: Arrowhead’s 2^nd^ annual cancer immunotherapy conference

**DOI:** 10.1186/2051-1426-2-6

**Published:** 2014-03-20

**Authors:** Adrian Bot, Maurizio Chiriva-Internati, Andrew Cornforth, Brian J Czerniecki, Soldano Ferrone, Kenneth Geles, Philip D Greenberg, Elaine Hurt, Richard C Koya, Masoud H Manjili, William Matsui, Richard A Morgan, Claudia M Palena, Daniel J Powell Jr, Nicholas P Restifo, David M Spencer, Raul Vizcardo, Albert J Wong, Lili Yang, John Yu

**Affiliations:** 1 Kite Pharma Inc., 2225 Colorado Avenue, Santa Monica, CA 90404, USA; 2 Texas Tech University of Health Sciences Center School of Medicine, Lubbock, TX, USA; 3 California Stem Cell, Inc., Irvine, CA, USA; 4 University of Pennsylvania, Philadelphia, PA, USA; 5 Harvard Medical School, BostonMA, USA; 6 Pfizer Inc., New york, NY, USA; 7 Fred Hutchinson Cancer Research Center, Seattle, WA, USA; 8 MedImmune, Gaithersburg, MD, USA; 9 Roswell Park Cancer Institute, Buffalo, NY, USA; 10 Virginia Commonwealth University School of Medicine, Massey Cancer Center, Richmond, VA, USA; 11 Johns Hopkins University School of Medicine, Baltimore, MD, USA; 12 National Cancer Institute, Bethesda, MD, USA; 13 Bellicum Pharmaceuticals, Inc, Houston, TX, USA; 14 RIKEN Research Center for Allergy and Immunology, Yokohama, Japan; 15 Stanford University Medical Center, Stanford, CA, USA; 16 University of California, Los Angeles, CA, USA; 17 Immunocellular Therapeutics, Calabasas, CA, USA

**Keywords:** Immunotherapy, Cancer stem cells, Hematopoietic stem cells, Adoptive T cell therapy, Antibodies, Vaccines

## Abstract

Investigators from academia and industry gathered on April 4 and 5, 2013, in Washington DC at the Arrowhead’s 2^nd^ Annual Cancer Immunotherapy Conference. Two complementary concepts were discussed: cancer “stem cells” as targets and therapeutic platforms based on stem cells.

## 

The hotly debated existence of cancer “stem cells” (CSC) or tumor-initiating cells (TIC) may have a tremendous impact on the future of cancer treatment. As modern oncology has thus far been largely unsuccessful in achieving long-term management of cancer, identifying and leveraging targets associated with tumor initiation, relapse or metastasis is a rationale course of action. In this regard, the field of oncology seems to be at an inflexion point: how do we identify and remove cancerous cells responsible for disease relapse, metastasis, and progression?

Unfortunately, CSCs seem to be quite refractory to available therapeutic strategies. Since immunotherapy offers mechanisms of action distinct from those of small molecules and perhaps more amenable to quiescent tumor cells, it has received significant interest as an alternate therapeutic approach. In addition, the understanding and utilization of normal hematopoietic and lymphoid stem cells could open alternate avenues to more effectively re-engineer and direct T cells against CSCs.

To discuss these aspects, investigators from academia and industry congregated in April 4 and 5 2013, in Washington DC at the Arrowhead’s 2^nd^ Annual Cancer Immunotherapy Conference, dedicated to Stem Cells and Cancer Immunotherapy. In brief, the theme of stem cells has been approached in two complementary ways:

● Cancer “stem cells” as a new category of targets for immune intervention, for both solid and hematologic malignancies.

● Therapeutic platforms based on stem cells: hematopoietic stem cells, reprogrammed pluripotent stem cells, stem cell-like memory T cells and tumor-derived stem cells.

This meeting was structured to start with research topics followed by talks by industry representatives and intermingled with workshop panels; however, this report presents the main ideas in a different flow to help frame the issues, discuss targets associated with cancer stem cells and then close with applicable platform technologies.

## Long term control of cancer and “cure” remain an unmet medical need

**Dr. William Matsui** (Johns Hopkins University School of Medicine) introduced elements of basic biology of cancer stem cells, utilizing Multiple Myeloma as an example. He conveyed the point that historically, the process of bringing new drugs to clinic and testing them is based on the expectation that such therapies need to show fairly rapid objective responses, manifested through tumor regression. Endpoints that require long term observation are being pursued only in late stage development, but most of the oncology therapies approved to date provide only incremental survival benefit despite some impressive objective response rates. Thus, the current drug development and regulatory strategies are geared for drugs that show rapid evidence of tumor shrinkage rather than substantially increased survival. Inasmuch as there may be differential sensitivity of tumor cell subsets with distinct biology within a cancerous process, this is a significant flaw in the process of drug development in oncology, since it could bias it away from therapies that obliterate tumor initiating cells or ‘cancer stem cells’. An alternative would be to test drug candidates on tumor cell subsets with increased clonogenic activity, which would accelerate the development of drugs that reduce relapse rates and prolong survival by removing the tumor-initiating cells. Dr. Matsui exemplified this model with findings of that CD19^+^ CD138^–^immunoglobulin-rearranged B cells carry the hallmarks of ‘tumor initiating cells’ in multiple myeloma, based on clonogenic analysis. This model of cancerous stem cells with B cell-like phenotype in multiple myeloma, led to trials that evaluated rituximab (anti-CD20 mAb) as add on to chemotherapy. The outcome of this evaluation raised the possibility that the CSCs in multiple myeloma are less differentiated (eg. CD19^+^ CD20^-^cells), or that myeloma cells carry intrinsic clonogenic properties.

## New targets expressed on tumour initiating cells are needed

Target identification and validation on “true” tumor-initiating cells has been a key bottleneck. Optimal targets should meet several characteristics: 1) expressed by cancerous cells; 2) expressed by tumorigenic cells (tumor-initiating cells or CSCs); 3) have a key role in tumor cell biology; and 4) not expressed in normal cells or tissues. In reality, the realm of the targets that meet these requirements is dismal, leading to significant efforts to identify new targets amenable to immune interventions.

One such target that meets all these criteria appears to be EGFRvIII, a truncated version of EGFR, endowed with autonomous signalling and pro-tumorigenic capabilities. Dr. Albert Wong from Stanford University Medical Center described this target and its biology. He summarized the evidence for EGFRvIII expression in CSCs in glioblastoma, utilizing the *in vitro* spherule assay. This supported the concept that in this disease, the cells with the highest tumorigenic properties are CD133 EGFRvIII double-positive cells. This renders EGFRvIII, a neoantigen devoid of expression in normal tissues, a quite appealing target for immune interventions. Nevertheless, EGFRvIII is neither homogenously expressed within tumors, nor it is indispensable as its expression could vary through various mechanisms during tumor progression. In addition EGFRvIII and other EGFR variants are expressed in various other tumor types. Currently, EGFRvIII is being pursued through vaccination aimed to elicit specific antibodies, through monoclonal antibodies and adoptive T cell therapy with genetically engineered T cells expressing EGFRvIII-directed chimeric antibody receptors (CARs).

**Dr. Philip Greenberg** from the University of Washington, Fred Hutchinson Cancer Research Center, presented two immunotherapeutic targets: a more recently described target Cyclin A1, and an earlier discovered target that received much attention to date, WT-1. He presented evidence of expression of Cyclin A1 and WT-1 in Acute Myeloid Leukemia (AML) CSCs, a cancer that is hierarchically organized and amenable to therapeutic intervention through targeting leukemogenic cells. These two intra-cellular targets are also expressed in other cancers including carcinomas. WT-1 is expressed at high levels and quite homogenously in many different cancers, but has some limited expression in normal cells such as stem cells. A TCR-based adoptive T cell therapy encompassing TCRs of certain affinities for the target MHC-peptide complex could endow the engineered T cell with abilities to recognize and affect cancerous rather than normal cells. Cyclin A1 is a new and exciting target: while the isoform Cyclin A2, derived from a closely related gene *CCNA2*, is ubiquitously expressed during mitosis in normal cells, expression of Cyclin A1 encoded by *CCNA1* is largely restricted to the meiotic phase in normal germinal cells but appears to be co-opted by many malignancies, including ~60% of cases of AML. T cells against Cyclin A1 and WT-1 epitopes were generated and tested in preclinical models. Since these targets are amenable to TCR-engineered adoptive T cell therapy, translational studies are already ongoing, with clinical evaluation in AML patients with antigen expressing leukemia and the appropriate HLA restricting element.

A target with a long research track record since its discovery, chondroitin sulfate proteoglycan 4 (CSPG4), was described by **Dr. Soldano Ferrone** (Massachusetts General Hospital and Harvard Medical School). He described the major characteristics of this complex and extensively glycosylated tumor antigen expressed on the cell membrane. Based on its expression profile, CSPG4 is potentially amenable to immune interventions such as antibody therapy and chimeric antigen receptor (CAR)-engineered T cells. CSPG4 is expressed on normal cells and highly up regulated on tumor cells of various origin: ectodermic, endodermic and mesodermic. Within tumors, CSPG4 could be also expressed on pericytes and other stromal cells, supporting a multi-pronged mechanism of action. The expression of CSPG4 on tumor initiating cells is of major interest as this could facilitate more potent immune interventions. CSPG4 expression on some normal cells associated with vasculature and central nervous system could be of concern; yet antibody based approaches that exploit possible differential post-translational modifications yielding specific tumor associated epitopes, could be a very fertile area of target identification and new drug development.

A target with tumor-specific membrane expression and thus amenable to antibody-based intervention is the oncofetal protein **5T4**. Normal expression of 5T4 also known as trophoblast glycoprotein (TPGB) is limited to placenta and embryonic stem cells. Expression of 5T4 is observed in many carcinomas, and notably, its over-expression in colorectal, gastric and ovarian cancers is associated with advanced disease and/or worse clinical outcome. 5T4 can function as a pro-migratory factor in embryonic cells that have undergone an epithelial-to-mesenchymal (EMT) transition and can also modulate CXCR4 and Wnt signalling. **Dr. Kenneth Geles** of Pfizer Inc. described the discovery that this well-known oncofetal protein is also enriched on cancer stem cells (tumor-initiating cells) in non-small cell lung carcinoma (NSCLC). In the H460 lung cancer cell line, the CD24^low^/CD44^high^ immunophenotype was determined to be the more tumorigenic subpopulation of cells and enriched for the 5T4 mRNA based on gene expression profiling. Additionally, sorting cells from a NSCLC patient derived xenograft (PDX) based solely on 5T4 expression confirmed that 5T4^high^ cells were indeed more tumorigenic than 5T4^low^ cells. In a primary NSCLC culture, 5T4 and markers of EMT were associated with an undifferentiated phenotype analogous to embryonic stem cells. Further, high levels of 5T4 expression were associated with poorly differentiated NSCLC tumors and worse overall survival. Interestingly, treatment of preclinical lung and breast cancer models with an anti-5T4 antibody drug conjugate (A1-mcMMAF) resulted in long-term tumor regressions, a finding that has been compelling enough to support the advancement of this novel therapy to Phase 1 clinical trials. This is the first proof-of-concept that targeting a heterogeneous subpopulation of cells at the apex of a cellular hierarchy with an ADC can inhibit tumor growth in preclinical models.

The biology of the target is critical as it could determine tumor escape mechanisms. Thus, targets associated with epithelial-mesenchymal transition (EMT) could be very valuable as they offer an opportunity to block tumor progression. **Dr. Claudia Palena** from the National Cancer Institute presented a relatively new target Brachyury that is a T-box transcription factor, intimately involved with EMT and associated with cancer cell stemness. Brachyury is an embryonically relevant protein required for the normal development of the mesoderm. While being absent in the majority of adult normal tissues, with the exception of low levels detected in normal testis and some thyroid tissues, Brachyury is aberrantly expressed in various tumor types, including lung, breast, colon and prostate carcinomas. Primary tumors and metastatic lymph nodes and distant metastasis of breast cancer have been shown to be highly positive for the expression of Brachyury protein; in addition, high levels of this transcription factor in primary breast tumors associate with poor prognosis in tamoxifen-treated patients. Expression of Brachyury in epithelial cancer cells drives the phenomenon of tumor EMT, a phenotypic switch characterized by the loss of epithelial markers, cell polarity and cell-to-cell contacts, and the simultaneous gain of mesenchymal markers, tumor cell motility, invasiveness and propensity to metastasize. Recent investigations have demonstrated that tumor cells undergoing EMT might also acquire features of stem-like cells, including the ability to survive cytotoxic therapies. In this regard, expression of Brachyury in lung and breast carcinoma cells has been associated with resistance to the cytotoxic effect of conventional anti-cancer modalities, including chemotherapy and radiation, both in vitro and in vivo. Due to its potentially relevant role in tumor resistance and metastasis, targeting of Brachyury is being investigated as a novel anti-tumor strategy. However, as a transcription factor, Brachyury cannot be targeted with monoclonal antibodies and the development of canonical small molecule inhibitors against this kind of molecules has been so far unsuccessful. It has been then proposed that a T-cell mediated immunotherapeutic approach could be a viable option to specifically eliminating tumor cells that express this transcription factor. In this regard, Brachyury has been shown to be an immunogenic molecule; an HLA-A0201 epitope was identified (WLLPGTSTL) and used to efficiently expand Brachyury-specific CD8^+^ T cells from the blood of cancer patients, in vitro. The expanded T-cell lines, more importantly, have been shown to lyse tumor cells that endogenously express Brachyury. Currently, various Brachyury-based cancer vaccine platforms are being developed.

HER-2/Neu is a well characterized cell surface cell growth receptor within the EGFR class of receptors, with amplified expression, pronounced biological role in tumors and strongly associated with breast and other carcinomas. **Dr. Brian Czerniecki** from the University of Pennsylvania described the expression of HER-2 in tumor cells with stem properties, with emphasis on luminal breast cancer. Such stem cell-like cancer cells co-expressing the estrogen receptor seem to have relatively low levels of HER-2 expression owing to mechanisms of up-regulation rather than gene amplification. Yet, they could be responsible for tumor relapse or progression. It is quite possible that such cells with rather dim expression levels of Her-2, could have a prominent role in tumor escape from targeted therapies irrespectively of the Her-2 status of the tumor. Since it is known that Her-2-directed antibody therapies are not applicable or not effective in patients with tumors that have modest levels of Her-2 expression, and that Her-2 positive patients treated with Herceptin are still at risk to relapse, alternate methods – such as immune interventions - to target such cells are needed. To be effective, these methods need to involve immune effectors that are capable to sense and react to low levels of antigen (eg. T cells generated *in vivo* through vaccination or generated *ex vivo*).

As mentioned above, another source of cancer stem cell-associated targets is represented by glioblastoma, one of the few solid tumors where there is quite convincing evidence in support of the existence of such cells, largely chemorefractory and radioresistant, and responsible for tumor relapse. **Dr. John Yu** of Immunocellular Therapeutics outlined the major characteristics of the brain cancer stem cells and discussed targets amenable to immune intervention against glioblastoma. Interestingly, as this cancer originates from the ectoderm, its shares quite a few antigens with melanoma. As these are tissue differentiation antigens or cancer testes antigens, with restricted expression within normal vital organs, they constitute a rich source of novel targets amenable to immunotherapy. An approach has been designed to target such antigens by utilizing active immunotherapy (therapeutic cancer vaccination) to elicit multiple immune responses against several targets that yield dominant epitopes expressed onto glioblastoma cells including cancer stem cells. One of the key limitations of such antigens is their intracellular expression, narrowing the panel of applicable immune interventions. It remains to be seen whether this concept could pave the way to a commonly utilized approach to define epitopes-derived from targets co-expressed by cancer stem.

**Dr. Elaine Hurt** from Medimmune described an *in vitro* high throughput assay utilized in their laboratory to discover novel targets associated with cancer stem cells, applicable to breast cancer and other tumor types. These targets are amenable to antibody based immunotherapy and related approaches, as they are expressed on the cell membrane. She discussed the rationale supporting **EZH2**, a novel target associated with the Wnt/Notch pathway, and thus closely related to stemness. This assay and screening methodology carries the promise of leading to identification of numerous membrane borne targets associated with tumor initiating cells.

An appealing class of targets are those with expression restricted to germinal cells and tumor tissues (**Cancer Testes Antigens**, CTA). Unfortunately, the CTAs heavily characterized to date are expressed inside the cell and thus are targetable only through immunotherapies that are directed at MHC-restricted epitopes, such as therapeutic vaccines and TCR-engineered T cells. A key question is whether there are CTAs with membrane expression also. **Dr. Maurizio Chiriva-Internati** from Texas Tech University presented emerging evidence that certain CTAs, associated with tumor initiating cells, can be also displayed onto the cell membrane. SP17, AKAP4, Ropporin, and PTTG1, recently and less characterized CTAs, present an increased interest since they are expressed in a broad range of tumors of widely different histological origin: multiple myeloma, lung cancer, ovarian and prostate carcinoma. AKAP4 in particular showed some promising evidence of membrane expression onto the multiple myeloma cells, by both flowcytometric analysis and immunohistochemistry. The possible expression of some CTAs onto the cell membrane, render these molecules promising targets for antibody and CAR-based approaches that are not MHC restricted. This should be an area of intense investigation as the number of targets identified to date and amenable to powerful immunotherapies such as CAR therapies, with expression strictly limited to tumor cells, is very narrow.

In all, while much more needs to be done, the prospect of identification of targets potentially associated with cancer stem cells and amenable to immunotherapy, is a catalyst to future discovery efforts and development of therapies that could afford more durable control of cancer.

## Therapeutic strategies must effectively target cancer stem cells

**Figure 1:**
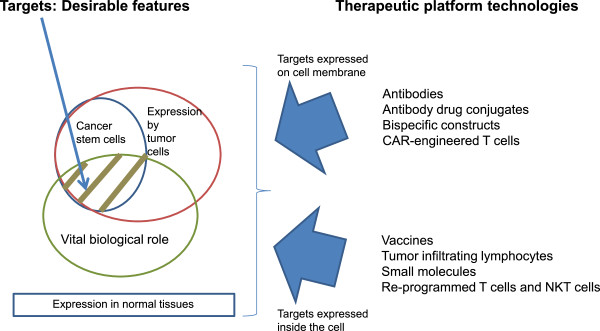
**Target characteristics and corresponding therapeutic approaches.** This diagram outlines the major desirable characteristics of new targets for immune interventions. Depending on expression pattern, such targets fall into two categories: 1) with membrane expression and thus targetable by antibodies, antibody drug conjugates, bispecific antibodies/constructs, CAR-engineered T cells and certain vaccines that induce antibodies; and 2) expressed inside the cells, and thus amenable to vaccines, tumor infiltrating lymphocytes, small molecules, re-programmed T cells and NKT cells.

The second element of a therapy against cancer stem cells, along with the appropriate target, is represented by the therapeutic platform which could be a biological or a small molecule capable to affect the viability or biological properties of such cells (Figure 1). Immune interventions are of particular interest as cancer stem cells or tumor initiating cells have a recognized refractoriness to conventional therapies (chemotherapy, radiotherapy) and small molecule targeted therapies. An immune mediated effect could be less dependent on the metabolic or proliferative state of the cells, and instead being linked to expression of target antigens and immune modulating receptors.

The last decade or so witnessed an unprecedented interest and progress with adoptive T cell therapy as a means to engineer a competent immune system, and thus render it capable to harness a cancerous process.

**Dr. Philip Greenberg,** from Fred Hutchinson Cancer Research Center, introduced and described the key elements of the adoptive T cell platform technology in all its diverse embodiments, each with their advantages and disadvantages. Three major elements must be considered to design safe and effective T cell therapies: 1) an appropriate antigenic target; 2) generation of a high avidity and high magnitude T cell response; and 3) the ability of tumor-reactive T cells to infiltrate and retain their function within the tumor microenvironment. Efforts to manipulate the TCR affinity and avidity by substituting amino acid residues within the complementary determining region 3 (CDR3) of the TCR chains, could lead to greatly improved **TCR-engineered T cells** from the point of view of efficacy. However, appropriate safeguards need to be pursued to pre-empt toxicities due to cross-reactivity against unintended targets. As methods to test for potential detrimental TCR cross-reactivity with cells within normal tissues have been limited and not adequately informative, this represents a hurdle for utilization of affinity enhanced TCRs and of TCRs generated in preclinical models to be utilized in clinic that requires the use of additional screening strategies. Other aspects of adoptive cell transfer with TCR-engineered T cells were also discussed: utilization of cytokines in the manufacturing process, T cell subsets and vaccines to enhance the effectiveness of adoptive T cell therapy and T cell subsets for enhanced adoptive T cell therapy. A cautionary note was provided relative to utilization of central memory T cells as a more globally efficacious T cell subset for immunotherapy, as there is emerging preclinical evidence that the effectiveness of T cell subsets could be dependent on the specific tumor that is targeted, probably due to the constellation of immune modulating receptors expressed within the tumor environment.

**Dr. Richard Morgan** from the National Cancer Institute presented the efforts of his group to design and test genetically engineered T cells encompassing chimeric antigen receptors (CARs) against antigens such as EGFRvIII and CSPG4. Such CARs are endowed with co-stimulatory signalling domains that provide the engineered T cells with supra-physiological capabilities. Dr. Morgan discussed their results obtained in a glioblastoma model, with cancer stem cells in the neurosphere assay as targets for an EGFRvIII-directed CAR engineered T cell approach. These newer CARs complement a growing pipeline that includes anti-CD19 CAR that showed objective clinical responses in leukemia and lymphoma. A third generation CAR against EGFRvIII, encompassing 4-1BB, CD28, CD3z as signalling domains, is currently undergoing phase 1 clinical testing in patients with relapsed glioblastoma multiformae.

**Dr. Daniel Powell** from the University of Pennsylvania presented his group’s efforts to design innovative T cell based therapies for cancer, based on lentiviral gene transfer to redirect human T cells against cancer antigens via **CARs**. He introduced an innovative design of an engineered T cell that could be armed *ex vivo* with the desired ligand for a specific tumor target or targeted against pre-labelled tumor cells *in vivo*, and is utilizable as a universal therapeutic platform. Dr. Powell showed the capacity of these universal immune receptor bearing T cells to target a multiple and diverse tumor antigens, including those expressed by cancer stem cells. The limitation of the approach could be the dilution of the receptor as the engineered T cells divide, however, this might also be viewed as a built in safety switch to prevent long term toxic effects of redirected T cells. Dr. Powell also discussed approaches to increase *on*-tumor immunity but limit *off*-tumor toxicity, by utilizing newer CAR T cell designs based on simultaneous recognition of multiple antigens. T cells co-expressing CARs that recognize distinct antigens and are linked to signalling motifs that would work efficiently in concert but not individually, could overcome a key limitation of currently available targets: leaky expression on certain normal cells. As an example, a dual CAR engineered T cell product co-recognizing mesothelin and folate receptor is being considered for testing in patients with ovarian carcinoma. Dr. Powell also discussed the significance of signalling domains selected for CAR design, and provided compelling preclinical evidence that the CD27 costimulatory domain is a viable option to other signalling domains such as the one borne by CD28. This work also demonstrated an important, but previously unappreciated, role for CD27 signalling in the formation of human T cell memory *in vivo*.

**Dr. Masoud Manjili** from Virginia Commonwealth University School of Medicine, Massey Cancer Center presented data relevant to the mechanism and applicability of adoptive cellular therapy (ACT), in preclinical models of breast carcinoma. He showed evidence that reprogramming tumor-sensitized immune cells in the presence of bryostatin 1/ionomycin and common gamma chain cytokines, *ex vivo*, generated memory T cells and activated NKT/NK cell populations. Presence of activated NKT/NK cells within the T cell product could have a major role to its activity, through interfering with the immune inhibiting role of the myeloid derived suppressor cells (MDSCs). A key limitation of the adoptive T cell immunotherapy technology is the relatively short life-span of functional T cells once they are infused into patients. Tumor-induced suppression of T cells is another barrier to effective immunotherapy of cancer. The use of activated NKT/NK cells along with canonical long-lasting memory T cells could tackle these two barriers. In addition, a considerable effort is spent on creating and utilizing renewable sources of T cells by tapping into the potential of normal hematopoietic and lymphoid stem cells.

In these lines, **Dr. Nicholas Restifo**, from the National Cancer Institute, described his group’s pioneering efforts in this area that led to discovery of stem cell-like memory T cells. Utilization of these cells for adoptive T cell therapy could overcome current limitations, such as reduced engraftment, limited persistence of T cells and need to preservation their functional capabilities over a longer timeframe. Dr. Restifo outlined several approaches to increase the yield of stem cell-like T cells in the manufacturing process of T cells for adoptive T cell therapy, by manipulating the Wnt signalling pathway or by utilizing certain cytokines that maintain the T cells in a juvenile state. This strategy could be applied to both tumor infiltrating lymphocytes (TILs) and receptor-engineered T cells alike. In addition, he shared late-breaking information on transcriptional and epigenetic programming of T cell stemness, which could be utilized to design approaches to de-differentiate such cells with *in vivo* renewable capabilities, from effector T cells isolated from tumors and other sources.

**Dr. Richard Koya** from University of California Los Angeles presented his team’s activities in support of generating and testing hematopoietic stem cells engineered with a TCR that recognizes NY-ESO-1, a well-known cancer testes antigen. They showed successful differentiation of functional CD8^+^ T cells in humanized mice. They so far successfully transduced human CD34+ cells with an average transduction efficiency of 50% (0.5 to 1 viral copies per cell). At three months post-transplant in a humanized NSG/HLA-A2 transgenic mouse model, CD8+ T cells harvested from spleens could expand *in vitro* and recognize NY-ESO-1 expressing targets, specifically killing melanoma cells. They also confirmed that a co-expressed PET reporter gene sr39TK was functional *in vivo*, as demonstrated by micro-PET imaging, and that ganciclovir administration could efficiently eliminate these cells. Dr. Koya outlined the plans to translate these findings to a clinical trial that integrates 1) stem cell transplantation with TCR-engineered hematopoietic stem cells, 2) adoptive cell therapy with TCR-engineered differentiated T cells, and 3) vaccination against NY-ESO-1. In essence, purified CD34+ cells from mobilized peripheral progenitor cells will be transduced with a codon-optimized lentiviral vector to induce expression of NY-ESO-1 TCR alpha and beta chains together with the PET marker/suicide gene sr39Thymidine Kinase. This novel clinical approach would allow continuous supply of T cells targeting a tumor antigen, addressing the issue seen when injected mature tumor-targeted T cells start vanishing from hosts by terminal differentiation.

**Dr. Lili Yang,** also from the University of California Los Angeles, discussed her team’s efforts to engineer immunity through the utilization of genetically engineered hematopoietic stem cells. She presented original findings based on an innovative imaging technology applied to T cells differentiated from TCR-engineered stem cells engrafted into mice. Dr. Yang shared tantalizing evidence that in this model, while differentiated CD8+ T cells migrate to lymph nodes where they expand and persist, the CD4+ T cells traffic to and persist within the lymphoid tissue associated with the intestinal tract. She also presented the roles of IL-15 and IL-7 in the homeostasis and survival of genetically engineered T cells that differentiate from TCR-engineered hematopoietic stem cells, to CD8+ and CD4+ T cells respectively.

In the current era of generation and manipulation of human induced pluripotent stem cells (iPS), there is significant and legitimate interest in understanding whether one can utilize re-programmed iPS as a renewable source of T cells for adoptive T cell therapy. **Dr. Raul Vizcardo** from RIKEN Research Center for Allergy and Immunology presented his group’s pioneering efforts to generate high numbers of tumor-antigen specific T cells from iPS de-differentiated from specific CD8^+^ T cells with proven anti-cancerous activity. Facilitated by techniques to separate the most desirable T cells from tumors, this approach can revolutionize TIL-based adoptive T cell therapy. Much more needs to be done to fully understand and appropriately tap into this technology as a renewable source of T cells with therapeutic potential, as well as to understand its safety profile influenced by the presence of undifferentiated cells.

Genetic cell engineering especially at the level of lymphoid, hematopoietic or pluripotent stem cells carries an inherent risk of oncogenesis. **Dr. David Spencer** from Bellicum Pharmaceuticals, Inc. introduced the CaspaCIDe™ system, which is a rapid, cell cycle-independent and non-immunogenic suicide gene that is triggered by the membrane-permeable, synthetic dimerizer ligand, AP1903. Clinical proof of principle has been demonstrated in a Phase I/II trial in the allogeneic, hematopoietic stem cell therapy setting. Potential use of CaspaCIDe to enable emerging stem cell therapies and tumor-targeting T cells was also discussed. CaspaCIDe comprises an FK506-binding protein 12 (FKBP12)-based, high affinity (K_d_ ~ 0.1 nM) ligand-binding domain fused to a truncated human Caspase-9 domain, lacking its Caspase-recruitment domain (CARD). In the presence of pM levels of AP1903, dimerization leads to its processing and initiation of apoptosis within 30 minutes. In the clinical setting symptoms of GVHD were alleviated in less than 24 hours after administration of AP1903. Outside of FKBP12v36 binding, the ligand is bio-inert and is currently delivered as a single intravenous infusion. This contrasts with the virally derived HSV-thymidine kinase (tk)/, ganciclovir (GCV) system that is already used to control some viruses, including cytomegalovirus, a major complication of bone-marrow transplants. The HSV-tk/GCV system is also immunogenic, inappropriate for immune competent hosts and relatively slow, requiring multiple infusions over several days for maximum efficacy on cycling cells. The other major, clinically promoted suicide gene therapy class, cell surface proteins, like CD20 or truncated EGFRt coupled with clinically approved antibodies, would be more limited by the diffusion characteristics of MoAbs and target endogenous marker^+^ cells as an unavoidable side effect.

A different platform technology that has been discussed comprises monoclonal antibodies that bind directly to tumour initiating cells**. Dr. Elaine Hurt** of Medimmune described some of her company’s efforts to discover cancer stem cell-associated targets suitable for antibody therapy. **Dr. Soldano Ferrone** of Harvard Medical School and **Dr. Kenneth Geles** from Pfizer Inc. presented their efforts on design, research and development of antibodies in context of select targets – CSPG4 and 5T4 respectively – for treatment of various solid tumours. More specifically, Dr. Geles described his team’s efforts to develop an antibody drug conjugate (ADC) against 5T4 comprising the tubulin inhibitor monomethylauristatin F, backed up by preclinical results in a lung cancer model that lead to a phase 1 clinical trial.

At last but not least, another platform technology discussed at this meeting was active immunotherapy, or therapeutic vaccines – spanning autologous dendritic cells (DC) and other types of vectors. This approach is aimed at inducing an immune response in the patient against targets associated with cancer stem cells. If successful, such an approach could be utilized to curb the capability of tumours to establish metastasis or relapse after standard treatment.

There were three DC-based therapeutic approaches discussed. **Dr. Brian Czerniecki** of the University of Pennsylvania described his group’s activities to develop a HER-2 pulsed DC1 vaccine that is effective in inducing CD4^+^, CD8^+^ T cell responses of T1 phenotype in 1) HER-2 high expressing ductal carcinoma in situ (DCIS) and early invasive breast cancer and 2) as well as intermediate expressing HER-2 early luminal cancers. Such a DC1 vaccine could eliminate HER-2 expressing DCIS cells in the HER-2 2^+^ population, compared with HER-2 high expressing early breast cancers. DC1 vaccines against HER-2 may be useful adjuvants to eliminate HER-2 expressing breast cancer stem cells that may be responsible for many late recurrences in patients with ER + luminal breast cancers. A trial to assess the DC1 vaccines in such patients after adjuvant therapy is being currently conducted.

**Dr. Andrew Cornforth,** of California Stem Cell, Inc. discussed the company’s progress utilizing their autologous stem cell based immunotherapy. This integrates an innovative method to generate stem cell-like cells from primary tumours, and utilization of autologous DC that are pulsed with cancer stem cell antigens. Dr. Cornforth discussed how the manufacturing challenges of autologous cell therapy products involving whole tumor cells as an antigen source have been overcome by utilizing proprietary techniques to purify and expand cancer stem cells. In addition, he presented two phase II clinical trials which demonstrated 5-year overall survival rates of 50% in stage IV metastatic melanoma patients with many patients experiencing prolonged periods of disease-free survival. Finally, he discussed the development of scale up and scale out technologies while conducting a large, multi-center phase III clinical trial.

**Dr. John Yu** of Immunocellular Therapeutics, presented the company’s focus on targeting brain cancer stem cell-associated antigens, shared with the neural crest, by utilizing peptide-pulsed DC vaccines. This program is in phase 2 clinical development following optimization of the vaccine formulation.

**Dr. Claudia Palena** from the NIH discussed in context of the target Brachyury, a Phase I clinical trial of a yeast recombinant vector encoding this protein, sponsored by Globimmune, which is currently ongoing in patients with advanced carcinomas. This vaccine platform, consisting of heat-killed recombinant *Saccharomyces cerevisiae* that expresses the full-length human Brachyury protein has been developed through a collaborative effort between the National Cancer Institute and GlobeImmune, and is currently undergoing Phase I clinical testing in patients with advanced carcinomas.

**Figure 2:**
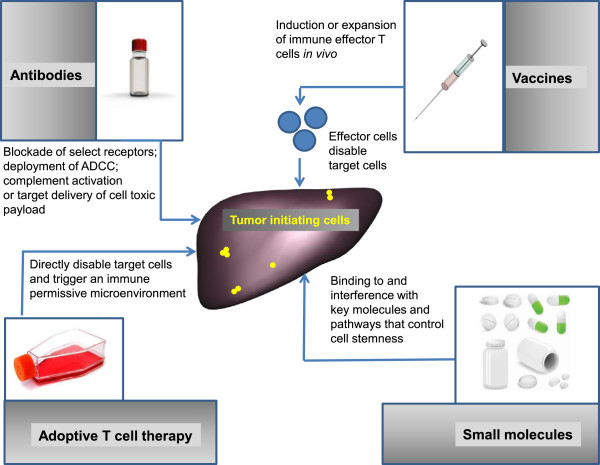
**Therapeutic approaches to target tumor-initiating or tumor-regenerating cells.** The diagram presents the four major categories of interventions potentially applicable: antibodies, vaccines, adoptive T cell therapy and small molecules.

As cancer stem cells are refractory to standard chemotherapy or radiotherapy, alternate immune based therapies could be a more effective means to eliminate these important cells and thereby attempt to control the process of cancer relapse, metastasis, and facilitating durable management of cancer. This is reflected by the diversity of immune-therapeutic approaches currently being tested to that aim, tailored to the characteristics of the targets (Figure 2).

## Closing remarks

In conclusion, the current drug development and regulatory strategies are geared for drugs that show rapid evidence of tumor shrinkage rather than substantially increased survival. Inasmuch as there may be differential drug sensitivity of tumor cell subsets with distinct biology within a cancerous process, this is a significant flaw in the process of drug development in oncology, since it could bias it away from therapies that obliterate tumor initiating cells or ‘cancer stem cells’. An alternative would be to test drug candidates on tumor cell subsets with increased clonogenic activity, which would accelerate the development of drugs that reduce relapse rates and prolong survival by removing the tumor-initiating cells. Dr. Matsui exemplified this model with findings of that CD19^+^ CD138^−^ immunoglobulin-rearranged B cells carry the hallmarks of ‘tumor initiating cells’ in multiple myeloma, based on clonogenic analysis. This model of cancerous stem cells with B cell-like phenotype in multiple myeloma, led to trials that evaluated rituximab (anti-CD20 mAb) as add on to chemotherapy. The outcome of this evaluation raised the possibility that the CSCs in multiple myeloma are less differentiated (eg. CD19^+^ CD20^−^ cells), or that myeloma cells carry intrinsic clonogenic properties.

We can infer that optimal targets should meet several characteristics: 1) expressed by cancerous cells; 2) expressed by tumorigenic cells (tumor-initiating cells or CSCs); 3) have a key role in tumor cell biology; and 4) not expressed in normal cells or tissues (Figure 1). In fact, the current realm of the targets that meet these requirements is dismal, leading to significant efforts to identify new targets amenable to immune interventions. As target identification and validation of expression by “true” tumor-initiating cells has been a key bottleneck, many speakers approached this subject, exemplifying it with specific candidates or novel discovery strategies (Table 1).

**Table 1:** Targets expressed on tumour initiating cells

**Targets**	**Speaker**	**Highlights**
EGFRvIII	Albert Wong, Stanford University Medical Center	• Truncated version of EGFR, with autonomous signalling and pro-tumorigenic capabilities
		• Expressed by CSCs in glioblastoma: the cells with the highest tumorigenic properties are CD133 EGFRvIII double-positive cells
		• Neither homogenously expressed within tumors, nor indispensable to tumor cells; its expression could vary during tumor progression
		• EGFRvIII is being pursued through vaccination, monoclonal antibodies and adoptive T cell therapy with CARs
Cyclin A1 Wilms Tumor antigen -1 (WT-1)	Philip Greenberg, University of Washington, Fred Hutchinson Cancer Research Center	• Cyclin A1 and WT-1 are expressed in Acute Myeloid Leukemia (AML) CSCs
		• Cyclin A1 is a new target: its expression encoded by *CCNA1* is largely restricted to the meiotic phase in normal germinal cells but appears to be co-opted by many malignancies, including ~60% of cases of AML
		• T cells against Cyclin A1 and WT-1 epitopes were generated and tested in preclinical models
		• Clinical evaluation of TCR-engineered adoptive T cell therapy is ongoing in AML patients with antigen^+^ leukemia and the appropriate HLA restricting element
Chondroitin sulfate proteoglycan 4 (CSPG4)	Soldano Ferrone, Massachusetts General Hospital and Harvard Medical School	• Complex and extensively glycosylated tumor antigen expressed on the cell membrane
		• Amenable to immune interventions such as antibody therapy and chimeric antigen receptor (CAR)-engineered T cells
		• Expressed on normal cells and highly upregulated on tumor cells of various origin: ectodermic, endodermic and mesodermic
		• Within tumors, CSPG4 could be also expressed on pericytes and other stromal cells, supporting a multi-pronged mechanism of action
		• CSPG4 is also expressed on tumor initiating cells
		• Its expression on some normal cells associated with vasculature and central nervous system could be of concern; yet antibody approaches directed to post-translational modifications could be a fertile area of new drug development
5T4 -trophoblast glycoprotein (TPGB)	Kenneth Geles, Pfizer Inc	• Tumor-specific membrane expression, amenable to antibody therapy. Normal expression of is limited to placenta and embryonic stem cells
		• 5T4 is over-expressed in colorectal, gastric and ovarian cancers and is associated with advanced disease and/or worse clinical outcome
		• It can function as a pro-migratory factor in embryonic cells that have undergone an epithelial-to-mesenchymal (EMT) transition and can also modulate CXCR4 and Wnt signalling
		• 5T4 is also enriched on cancer stem cells (tumor-initiating cells) in non-small cell lung carcinoma (NSCLC)
		• In the H460 lung cancer cell line, the CD24^low^/CD44^high^ subset is most tumorigenic and enriched for the 5T4 mRNA
		• Sorting cells from a NSCLC patient derived xenograft (PDX) based on 5T4 expression confirmed that 5T4^high^ cells were more tumorigenic
		• High levels of 5T4 expression were associated with poorly differentiated NSCLC tumors and worse overall survival
		• Treatment of preclinical lung and breast cancer models with an anti-5T4 antibody drug conjugate (A1-mcMMAF) resulted in long-term tumor regressions. This is the first proof-of-concept targeting a heterogeneous subpopulation of cells at the apex of a cellular hierarchy with an ADC
		• This therapeutic candidate entered Phase 1 clinical trials
Brachyury	Claudia Palena, National Cancer Institute	• Brachyury is an embryonically relevant T-box protein required for the normal development of the mesoderm
		• It is aberrantly expressed in various tumor types, including lung, breast, colon and prostate carcinomas • Primary tumors, metastatic lymph nodes
		• and distant metastasis of breast cancer have been shown to be highly positive for Brachyury
		• Expression of Brachyury in epithelial cancer cells drives EMT. Tumor cells undergoing EMT acquire features of stemness
		• Expression of Brachyury in lung and
		• breast carcinoma cells has been associated with resistance to conventional anti-cancer modalities
		• As antibodies are not applicable and small molecules have been unsuccessful, T-cell mediated immunotherapeutic approaches against Brachyury are being developed
		• Brachyury has been shown to be an immunogenic molecule; an HLA-A0201 epitope was identified (WLLPGTSTL) and used to efficiently expand Brachyury-specific CD8+ T cells from patients
		• Brachyury-based cancer vaccine is in clinical development
HER-2/Neu	Brian Czerniecki, University of Pennsylvania	• Well characterized cell surface, cell growth receptor within the EGFR class of receptors, with amplified expression and prominent biology
		• Expressed on CSC in luminal breast cancer
		• When co-expressed with the estrogen receptor, HER-2 expression is up-regulated by mechanisms other than gene amplification
		• CSC with dim Her-2 expression could have a role in tumor escape from targeted therapies, irrespectively of the Her-2 status of the primary tumor
		• Her-2-directed antibody therapies are not applicable or not effective in patients with tumors that have modest levels of Her-2 expression
		• Thus, alternate immune interventions to target such cells are needed, such as therapeutic vaccines, currently in development
EZH2	Elaine Hurt, Medimmune	• Described an *in vitro* high throughput assay to discover novel targets associated with cancer stem cells
		• These targets are amenable to antibody based immunotherapy and related approaches, as they are expressed on the cell membrane
		• Described EZH2, a novel target associated with the Wnt/Notch pathway, and thus closely related to stemness
Cancer testes antigens (CTAs)	John Yu, Immunocellular Therapeutics	• Another source of cancer stem cell-associated targets is represented by glioblastoma, with convincing evidence in support of their existence
		• These cells are largely chemorefractory and radioresistant, and responsible for tumor relapse
		• As this cancer originates from the ectoderm, its shares antigens with melanoma. CTAs with restricted expression within normal vital organs, constitute a rich source of novel targets amenable to immunotherapy
		• An approach has been designed to target such antigens by utilizing active immunotherapy (therapeutic cancer vaccination) to elicit multiple immune responses against glioblastoma cancer stem cells
	Maurizio Chiriva-Internati, Texas Tech University	• Most CTAs characterized to date are expressed inside the cell and thus are targetable only through immunotherapies that are directed at MHC-restricted epitopes (therapeutic vaccines and TCR-engineered T cells)
		• There is emerging evidence that certain CTAs, associated with tumor initiating cells, can be also displayed onto the cell membrane
		• Such targets are SP17, AKAP4, Ropporin, and PTTG1, expressed in a broad range of tumors of widely different histological origin: multiple myeloma, lung cancer, ovarian and prostate carcinoma
		• AKAP4 in particular showed some promising evidence of membrane expression in multiple myeloma cells
		• The possible expression of some CTAs onto the cell membrane, render these molecules promising targets for antibody and CAR-approaches

The second element of a therapy against cancer stem cells, along with the appropriate target, is represented by therapeutic platforms which could be biologicals or small molecules capable to affect the viability or biological properties of such cells (Figure 2). Immune interventions are of particular interest as cancer stem cells or tumor initiating cells have a recognized refractoriness to conventional therapies (chemotherapy, radiotherapy) and small molecule targeted therapies. An immune mediated effect could be less dependent on the metabolic or proliferative state of the cells, and instead being linked to expression of target antigens and immune modulating receptors. In light of the importance of this topic, many of the speakers at this event reviewed and discussed a broad range of immune therapeutic platforms including but not limited to approaches based on normal lymphoid stem cells (Table 2).

**Table 2:** Therapeutic strategies to target cancer stem cells

**Category**	**Speaker**	**Highlights**
T cell receptor (TCR) engineered T cells	Philip Greenberg, Fred Hutchinson Cancer Research Center	• Three major elements must be considered to design safe and effective T cell therapies: 1) an appropriate antigenic target; 2) generation of a high avidity and high magnitude T cell response; and 3) the ability of tumor-reactive T cells to infiltrate and retain their function within the tumor microenvironment
		• Efforts to manipulate the TCR affinity and avidity by substituting amino acid residues within the complementary determining region 3 (CDR3) of the TCR chains, could lead to greatly improved TCR-engineered T cells from the point of view of efficacy
		• However, appropriate safeguards need to be pursued to pre-empt toxicities due to cross-reactivity against unintended targets. This represents a hurdle for utilization of affinity enhanced TCRs and of TCRs generated in preclinical models
		• Other aspects of adoptive cell transfer with TCR-engineered T cells were also discussed: utilization of cytokines in the manufacturing process, T cell subsets and vaccines to enhance the effectiveness of adoptive T cell therapy and T cell subsets for enhanced adoptive T cell therapy
		• Cautionary note was provided on using central memory T cells, as there is emerging preclinical evidence that the effectiveness of T cell subsets could be dependent on the type of targeted tumor
Chimeric antibody receptor (CAR)-engineered T cells	Richard Morgan, National Cancer Institute	• Presented efforts to design and test genetically engineered T cells encompassing chimeric antigen receptors (CARs) against antigens such as EGFRvIII and CSPG4
		• Such CARs are endowed with co-stimulatory signalling domains that provide the engineered T cells with supra-physiological capabilities • Discussed results obtained in a glioblastoma model, with cancer stem cells in the neurosphere assay as targets for an EGFRvIII-directed CAR engineered T cell approach
		• A third generation CAR against EGFRvIII, encompassing 4-1BB, CD28, CD3z as signalling domains, is currently undergoing phase 1 clinical testing in patients with glioblastoma multiformae
	Daniel Powell, University of Pennsylvania	• Introduced an innovative design of an engineered T cell that could be armed *ex vivo* with the desired ligand for a specific tumor target or targeted against pre-labelled tumor cells in vivo (universal therapeutic platform)
		• Such universal immune receptor bearing T cells can target multiple and diverse tumor antigens, including those expressed by cancer stem cells
		• The limitation of the approach could be the dilution of the receptor as the engineered T cells divide. However, this might also be viewed as a built in safety switch to prevent long term toxic effects of redirected T cells
		• Also discussed approaches to increase on-tumor immunity but limit off-tumor toxicity, by utilizing newer CAR T cell designs based on simultaneous recognition of multiple antigens
		• As an example, a dual CAR engineered T cell product co-recognizing mesothelin and folate receptor is being considered for testing in patients with ovarian carcinoma
		• Discussed the significance of signalling domains selected for CAR design, and provided compelling preclinical evidence that the CD27 costimulatory domain is a viable option
Adoptive T + NKT/NK cell therapy	Dr. Masoud Manjili, Virginia Commonwealth University School of Medicine, Massey Cancer Center	• Presented data relevant to the mechanism and applicability of adoptive cellular therapy (ACT) in preclinical models of breast carcinoma.
		• Showed evidence that reprogramming tumor-sensitized immune cells in the presence of bryostatin 1/ionomycin and common gamma chain cytokines, generated memory T cells and activated NKT/NK cell populations
		• Presence of activated NKT/NK cells within the T cell product could have a major role, through interfering with immune inhibition by the myeloid derived suppressor cells (MDSCs)
		• The use of activated NKT/NK cells along with canonical long-lasting memory T cells could prolong the functionality of T cells and overcome inhibiting mechanisms
Stem cell like memory T cells for adoptive T cell therapy	Nicholas Restifo, National Cancer Institute	• Described his group’s pioneering efforts in this area that led to discovery of stem cell-like memory T cells
		• Utilization of these cells for adoptive T cell therapy could overcome current limitations, such as reduced engraftment, limited persistence of T cells and need to preservation their functional capabilities over a longer timeframe
		• Outlined several approaches to increase the yield of stem cell-like T cells in the manufacturing process of T cells for adoptive T cell therapy, by manipulating the Wnt signalling pathway or by utilizing certain cytokines that maintain the T cells in a juvenile state
		• Shared late-breaking information on transcriptional and epigenetic programming of T cell stemness, which could be utilized to design approaches to de-differentiate such cells with in vivo renewable capabilities, from effector T cells isolated from tumors and other sources
Hematopoietic stem cells	Richard Koya, University of California Los Angeles	• Presented generation and testing of hematopoietic stem cells engineered with a TCR that recognizes NY-ESO-1
		• Showed successful differentiation of functional CD8+ T cells in humanized mice, from transduced human CD34+ cells with an average transduction efficiency of 50%
		• At three months post-transplant, CD8+ T cells harvested from spleens could expand in vitro and recognize NY-ESO-1 expressing targets, specifically killing melanoma cells
		• Also confirmed that a co-expressed PET reporter gene sr39TK was functional in vivo, as demonstrated by micro-PET imaging; ganciclovir administration could efficiently eliminate these cells
		• Outlined plans to translate these findings to a clinical trial that integrates 1) stem cell transplantation with TCR-engineered hematopietic stem cells, 2) adoptive cell therapy with TCR-engineered differentiated T cells, and 3) vaccination
		• In this trial, purified CD34+ cells from mobilized peripheral progenitor cells are transduced with a codon-optimized lentiviral vector to induce expression of NY-ESO-1 TCR alpha and beta chains together with the PET marker/suicide gene
	Lili Yang, University of California Los Angeles	• Discussed her team’s efforts to engineer immunity through the utilization of genetically engineered hematopoietic stem cells
		• Presented results of an innovative imaging technology applied to T cells differentiated from TCR-engineered stem cells engrafted into mice. Showed evidence that in this model, while differentiated CD8+ T cells migrate to lymph nodes where they expand and persist, the CD4+ T cells traffic to and persist within the lymphoid tissue associated with the intestinal tract
		• Discussed the roles of IL-15 and IL-7 in the homeostasis and survival of genetically engineered T cells that differentiate from TCR-engineered hematopoietic stem cells, to CD8+ and CD4+ T cells respectively
Human induced pluripotent stem cells (iPS)	Raul Vizcardo, RIKEN Research Center for Allergy and Immunology, Japan	• In the current era of generation and manipulation of, there is significant and legitimate interest in understanding whether one can utilize re-programmed iPS as a renewable source of T cells for adoptive T cell therapy
		• Presented pioneering efforts to generate high numbers of tumor-antigen specific T cells from iPS de-differentiated from specific CD8+ T cells with proven anti-cancerous activity. Facilitated by techniques to separate the most desirable T cells from tumors, this approach can revolutionize TIL-based adoptive T cell therapy
		• Much more needs to be done to fully understand and appropriately tap into this technology as a renewable source of T cells with therapeutic potential, as well as to understand its safety profile influenced by the presence of undifferentiated cells
CaspaCIDe™ system (applicable to adoptive cell therapy)	David Spencer, Bellicum Pharmaceuticals Inc.	• Genetic cell engineering especially at the level of lymphoid, hematopoietic or pluripotent stem cells carries an inherent risk of oncogenesis
		• CaspaCIDe™ system is a rapid, cell cycle-independent and non-immunogenic suicide gene that is triggered by the membrane-permeable, synthetic dimerizer ligand, AP1903
		• Clinical proof of principle has been demonstrated in a Phase I/II trial in the allogeneic, hematopoietic stem cell therapy setting
		• Potential use of CaspaCIDe to enable emerging stem cell therapies and tumor-targeting T cells was also discussed
		• CaspaCIDe comprises an FK506-binding protein 12 (FKBP12)-based, high affinity (Kd ~ 0.1 nM) ligand-binding domain fused to a truncated human Caspase-9 domain, lacking its Caspase-recruitment domain (CARD). In the presence of pM levels of AP1903, dimerization leads to its processing and initiation of apoptosis within 30 minutes
		• In the clinical setting symptoms of
		• GVHD were alleviated in less than 24 hours after administration of AP1903
		• Outside of FKBP12v36 binding, the ligand is bio-inert and is currently delivered as a single intravenous infusion
		• This contrasts with the virally derived HSV-thymidine kinase (tk)/, ganciclovir (GCV) system that is immunogenic, inappropriate for immune competent hosts, and relatively slow, requiring multiple infusions over several days for maximum efficacy on cycling cells
		• The other major suicide gene therapy class, cell surface proteins, like CD20 or truncated EGFRt coupled with clinically approved antibodies, would be more limited by the diffusion characteristics of MoAbs and possible co-targeting of normal cells
Monoclonal antibodies and antibody drug conjugates	Elaine Hurt, Medimmune	• Described some of her company’s efforts to discover cancer stem cell-associated targets suitable for antibody therapy
	Soldano Ferrone, Harvard Medical School	• Presented efforts on design, research and develop antibodies against CSPG4 for treatment of various solid tumours
	Kenneth Geles, Pfizer Inc.	• Described his team’s efforts to develop an antibody drug conjugate (ADC) against 5T4, comprising the tubulin inhibitor monomethylauristatin F, backed up by preclinical results in a lung cancer model that lead to a phase 1 clinical trial
Therapeutic cancer vaccines	Brian Czerniecki, University of Pennsylvania	• Described his group’s activities to develop a HER-2 pulsed DC1 vaccine that is effective in inducing CD4, CD8 T cell responses of T1 phenotype in 1) HER-2 high expressing ductal carcinoma in situ (DCIS) and early invasive breast cancer and 2) as well as intermediate expressing HER-2 early luminal cancers
		• Such a DC1 vaccine could eliminate HER-2 expressing DCIS cells in the HER-2 2^+^ population, compared with HER-2 high expressing early breast cancers
		• DC1 vaccines against HER-2 may be useful to eliminate HER-2 expressing breast cancer stem cells that may be responsible for many late recurrences in patients with ER + luminal breast cancers
		• A trial to assess the DC1 vaccines in such patients after adjuvant therapy is being currently conducted
	Andrew Cornforth, of California Stem Cell, Inc.	• Discussed the company’s progress utilizing their autologous stem cell based immunotherapy. This integrates an innovative method to generate stem cell-like cells from primary tumours, and utilization of autologous DC that are pulsed with cancer stem cell antigens
		• Also discussed how the manufacturing challenges of autologous cell therapy products involving whole tumor cells as an antigen source have been overcome by utilizing proprietary techniques to purify and expand cancer stem cells
		• Presented two phase II clinical trials which demonstrated 5-year overall survival rates of 50% in stage IV metastatic melanoma patients
		• Discussed the development of scale up and scale out technologies while conducting a large, multi-center phase III clinical trial
	John Yu, Immunocellular Therapeutics Inc	• Presented the company’s focus on targeting brain cancer stem cell-associated antigens, shared with the neural crest, by utilizing peptide-pulsed DC vaccines
		• This program reached phase 2 clinical development following optimization of the vaccine formulation
	Claudia Palena, NIH	• Discussed in context of the target Brachyury, a Phase I clinical trial of a yeast recombinant vector encoding this protein, sponsored by Globimmune, in patients with carcinoma
		• This vaccine platform, consisting of heat-killed recombinant Saccharomyces cerevisiae has been developed through a collaborative effort between the National Cancer Institute and GlobeImmune

In conclusion, the concept of cancer stem cells remains hotly debated but could be a fertile ground for drug development, to address cancer resistance, relapse and progression. Irrespectively of semantics and applicability of the concept in a stricter or more relaxed fashion across all tumor types, there seems to be clear that the target cells of interest meet three key criteria:

1) Are relatively refractory to current therapies such as chemotherapies, radiotherapy and small molecule targeted therapies;

2) Can persist in the body for prolonged intervals in a stealth mode, without being subjected to immune surveillance or other homeostatic mechanisms;

3) Could re-generate the entire tumoral process usually in an evolved, more clinically aggressive mode.

**Figure 3:**
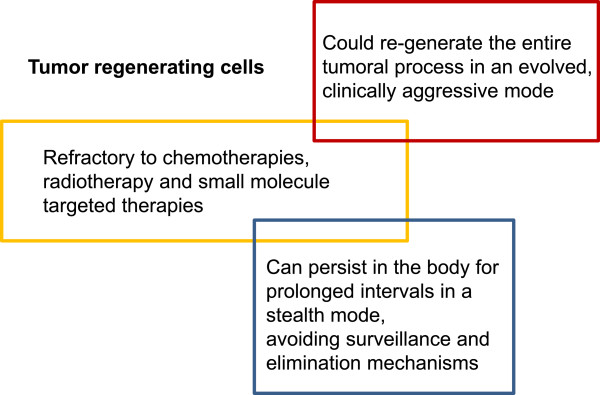
Major characteristics of tumor-regenerating cells, a category of “cancer stem cells” that should be the focus of research and development towards long term management of cancer.

Based on these characteristics, we advance the concept of “tumor regenerating cells” (TRC), as the subset of residual and resilient cancerous cells that mediate the process of relapse and tumor progression (Figure 3). These are the very target cells that should be in the cross-hairs of drug development for cancer. They may share features with, but do not need to recapitulate each and every characteristics of stem cells.

Last but not least, industry started to take a keen interest in cell based immune interventions to treat cancer due to the prospect of objective and durable clinical responses. Key, but addressable hurdles are the complex biology and manufacturing of such therapeutics. Thus, the longer term questions are how to define targets specifically associated with TRC, and how to optimize immunotherapies to effectively eliminate those cells thus achieving durable response or functional cure.

## Competing interests

A.B., A.C., K.G., E.H., D.S. and J.Y. are employees or in the management of pharmaceutical or biotech companies, receiving salary, compensation and/or having stock ownership.

## Authors’ contributions

All authors contributed equally to the event and manuscript writing. AB structured and drafted the manuscript, and designed the diagrams. All authors read and approved the final manuscripts.

